# Perioperative Implications in a Hypertrophic Cardiomyopathy Patient Undergoing Endonasal Orbital Decompression: A Case Report

**DOI:** 10.7759/cureus.67115

**Published:** 2024-08-18

**Authors:** Priyanka D Tikait, Ridhima Sharma, Anu Kewlani, Chayanika Kutum, Ripon Choudhary

**Affiliations:** 1 Department of Anaesthesiology, All India Institute of Medical Sciences, Nagpur, Nagpur, IND; 2 Department of Anaesthesiology, Maulana Azad Medical College, New Delhi, IND

**Keywords:** case report, sphenopalatine block, perioperative period, hyperthyroidism, graves ophthalmopathy

## Abstract

Thyroid eye disease (TED) is an autoimmune disease of the retro-ocular tissue and occurs in patients with Graves’ disease with hyperthyroidism. In severe cases, it can lead to vision loss; hence, timely management is important. Hypertrophic cardiomyopathy (HCM) is one of the systemic manifestations related to hyperthyroidism. We present a case of successful perioperative management of Graves’ disease with TED and HCM posted for endonasal decompression surgery. Hyperthyroidism itself has many implications on perioperative anesthetic management, which can become more complicated when associated with co-existing HCM. However, our case proves that detailed preoperative evaluation, vigilant intraoperative monitoring, and adequate analgesia can lead to a smooth perioperative course for HCM patients for non-cardiac surgeries.

## Introduction

Thyroid eye disease (TED) is an autoimmune condition frequently associated with hyperthyroidism due to Graves’ disease. In severe cases, the expansion of the orbital contents and fibrosis can lead to optic nerve compression and vision loss, which may require surgical decompression [1]. The available approaches for surgical orbital decompression are transcaruncular, transconjunctival, and endoscopic transnasal [1]. The endoscopic approach has the added advantage for the surgeons by providing a better view of the posterior ethmoidal sinuses and the sphenoid sinuses for posterior decompression and provides the opportunity to decompress the optic nerve if the need arises [2]. Ideally, patients should be euthyroid and have been in the dormant phase of TED for a minimum of six months before undergoing ophthalmic surgical intervention. Hyperthyroid patients requiring surgery and anesthesia face risks and complications in the perioperative period due to deranged thyroid hormone status and its systemic effects, including change in anesthetic drug requirement, risk of thyroid storm, hyperthermia, sympathetic surge, etc. Intraoperative manipulation of the globe poses a risk of oculocardiac reflex. Hypertrophic cardiomyopathy (HCM) is one of the rare extrathyroidal manifestations of Graves’ disease. If present, it can pose additional risk to the already compromised hyperthyroid patient [3]. Thyroid hormones have a direct effect on the cardiac myocytes and induce a cardiac hypertrophy phenotype, resulting in HCM. The major pathophysiology in HCM includes a dynamic obstruction of the left ventricular outflow tract (LVOT) due to mitral valve systolic anterior motion, diastolic dysfunction, and reduced coronary vasodilator reserve. These factors make patients with HCM prone to myocardial ischemia and arrhythmias (e.g., atrial fibrillation and ventricular tachycardia/fibrillation). The perioperative stress of anesthesia and surgery may trigger or augment the dynamic LVOT and may lead to grave hemodynamic complications mentioned above. Anesthesiologists should be vigilant to quickly recognize the risk factors, respond acutely, and reverse the potential complications resulting from the interaction of perioperative stress with HCM.

Here, we describe the successful perioperative management of a case of Graves’ disease-related TED with HCM posted for bilateral endonasal decompression.

## Case presentation

**Figure 1:**
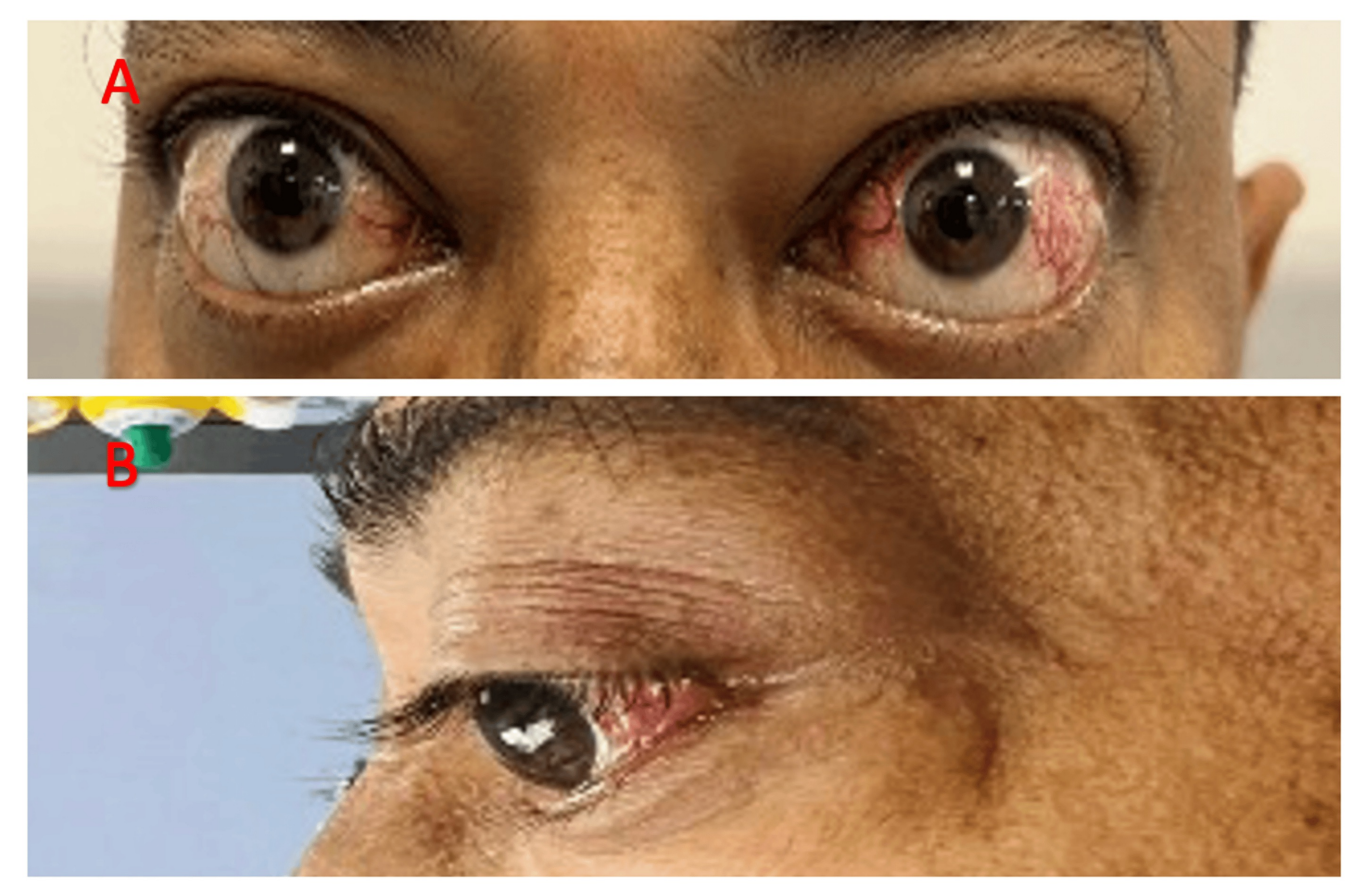
A 43-year-old male with Graves’ disease-related thyroid eye disease. (A) Frontal view. (B) Lateral view showing bilateral eye proptosis.

The consent for writing the case report was obtained along with the surgical consent from the patient. We report a case of a 43-year-old male presenting with a history of bilateral eye proptosis and blurring of vision in the left eye for two months (Figure 1).

The patient was a known case of Graves’ disease with hyperthyroidism for five years, for which he was on carbimazole 10 mg tablet twice a day. He was also a known hypertensive on bisoprolol 5 mg tablet once a day. The patient had a history of three episodes of cerebrovascular accidents (CVAs) in the past five years for which he was on tablet aspirin 75 mg and clopidogrel 75 mg. The last episode of CVA was four months back resulting in right-sided hemiplegia, which recovered over time. However, there was no history of atrial fibrillation, any cardiac clot, or deep venous thrombosis. The patient also complained of atypical chest pain and occasional palpitations. His metabolic equivalent of task score (METs) was less than 4. Systemic examination revealed a systolic murmur of crescendo-decrescendo type audible between the left sternal border and apical area. The rest of the general and systemic examination revealed no positive findings.

**Figure 2:**
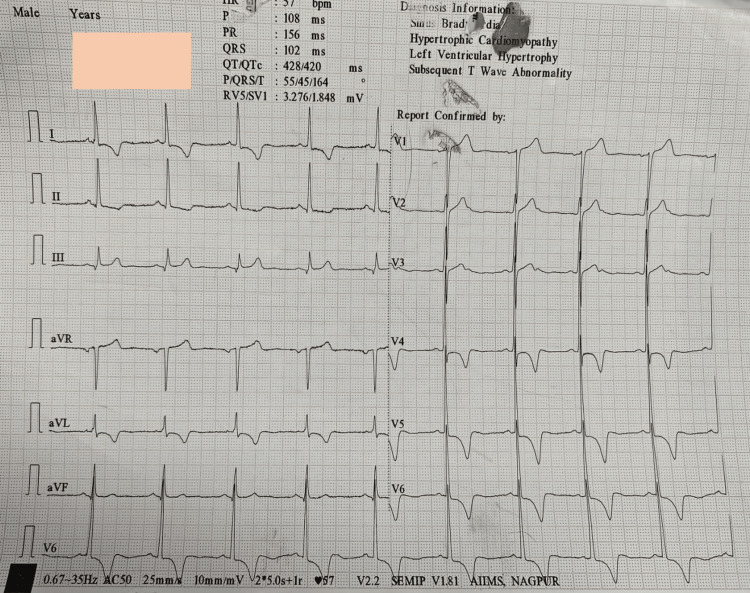
ECG showing ST segment sagging pattern in leads I, II, aVL, and V4-V6 with left ventricular hypertrophy. aVL: augmented vector left.

The patient was planned for bilateral endonasal decompression for which he was further investigated. Routine blood investigations, coagulation profiles, and thyroid function tests (TFT) were within normal limits. Chest X-ray showed cardiomegaly with clear lung fields. ECG was suggestive of ST segment sagging in leads I, II, aVL (augmented vector left), and V4-V6, with left ventricular hypertrophy (LVH) (Figure 2).

**Figure 3:**
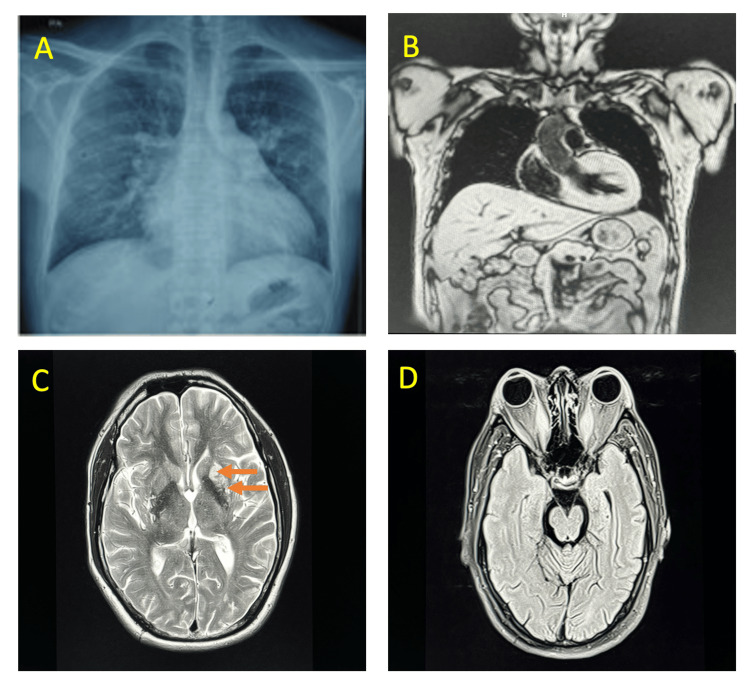
Investigations of the patient. (A) Chest X-ray posteroanterior view revealing cardiomegaly. (B) Cardiac MRI study revealing asymmetrical hypertrophic cardiomyopathy. (C) MRI of the brain, axial cuts (arrows) showing chronic lacunar infarcts in left centrum semiovale and corona radiate. (D) MRI orbits showing enlargement of bilateral recti muscles with resultant bilateral proptosis.

2D echocardiography revealed an ejection fraction (EF) of 60% and asymmetrical septal hypertrophy with severe LVH. Apical hypertrophy was noted with complete obliteration of the left ventricular (LV) cavity. A cardiac MRI was also done, which showed asymmetrical thickening of the cardiac wall involving basal, mid anterior, anteroseptal, and mid inferoseptal segments with maximum end-diastolic thickness measuring 22 mm in the basal anterior segment. There was flow acceleration and mid-ventricular cavity obliteration in end systole with a left ventricle ejection fraction of 67%. CT coronary angiography (CAG) was suggestive of 75% narrowing of the proximal left circumflex artery, 65% narrowing of the proximal left anterior descending artery, and 50% narrowing of the right coronary artery during systole. LVOT gradient was 9 mmHg (peak) and 5 mmHg (mean). MRI of the brain showed chronic infarcts in the left centrum semiovale and corona radiate region (Figure 3).

**Figure 4:**
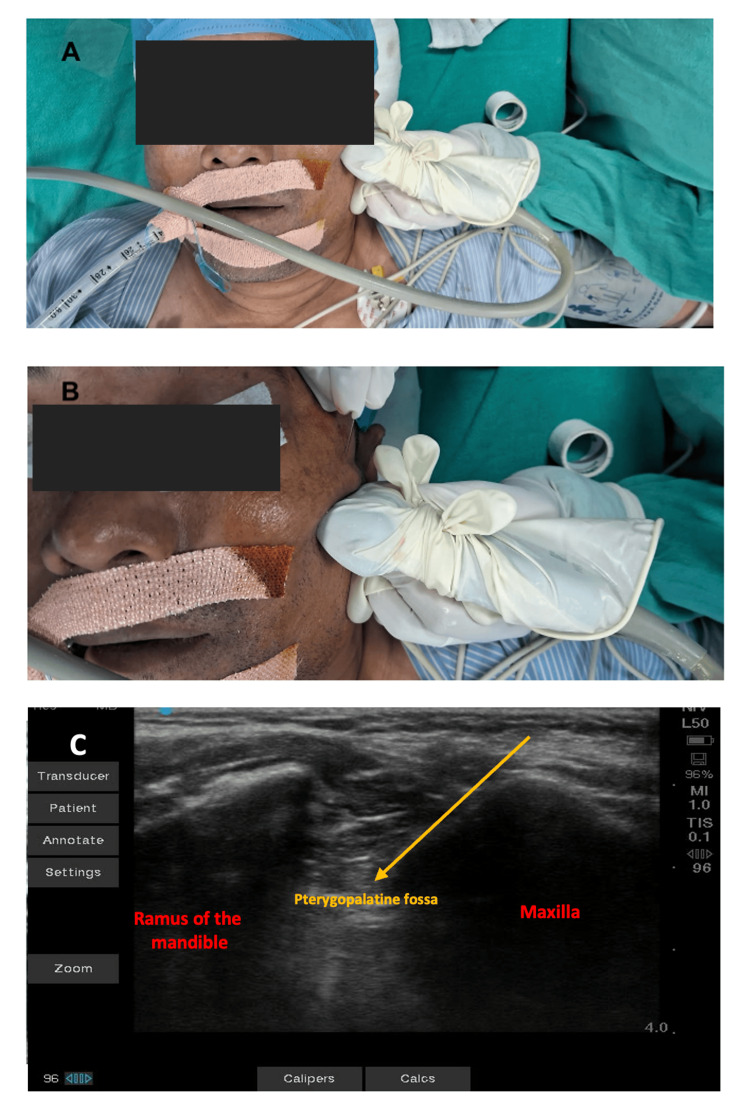
Real-time ultrasound-guided sphenopalatine ganglion blockade. (A) Placement of linear ultrasound probe inferior and parallel to the zygomatic process. (B) Block needle inserted just behind the posterior orbital rim and above the zygomatic process, directing it 45° caudally. (C) Corresponding ultrasound image to perform the sphenopalatine ganglion block; local anesthetic injected into the pterygopalatine fossa.

Clopidogrel was stopped five days prior to surgery. Antihypertensive and antithyroid drugs were continued till the morning of surgery. In the operating room, all American Society of Anesthesiologists (ASA) standard monitors were attached (pulse oximetry, non-invasive blood pressure, ECG, neuromuscular train of four monitors, and bispectral index monitor). Baseline vitals were within normal limits. The patient was induced with fentanyl, etomidate, and rocuronium. To prevent the intubation response, esmolol 30 mg was given 90 seconds before intubation, followed by gentle laryngoscopy and tracheal intubation. The left radial artery and USG-guided right subclavian vein catheters were inserted. General anesthesia was maintained with sevoflurane, oxygen, and air. Normothermia was maintained with a warmed-air blanket. Under all aseptic precautions, a USG-guided bilateral sphenopalatine block was administered to the patient with bupivacaine 0.25% 10 ml given on each side after negative aspiration (Figure 4).

**Figure 5:**
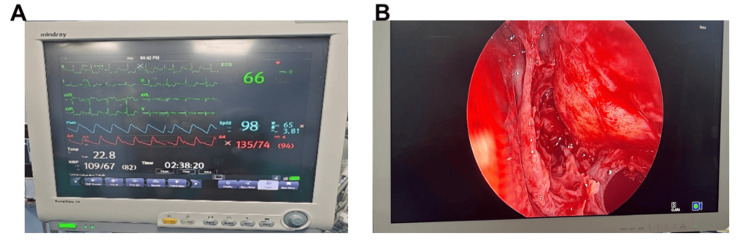
(A) Monitor showing stable intraoperative hemodynamics. (B) A relatively clean surgical field seen on the endoscope monitor.

The hemodynamics remained stable at the incision and even throughout the surgery (Figure 5).

There was no episode of significant hypotension, hypertension, or tachycardia intraoperatively. Central venous pressure remained between 8 and 10 mmHg. Blood loss was minimal. The surgery lasted for four hours. At the end of the surgery, anesthesia was reversed and the patient's trachea was extubated. Esmolol 30 mg was administered again to prevent exaggerated hemodynamic response during extubation. Following extubation, the patient was hemodynamically stable and shifted to ICU for observation and further management. In the ICU, hemodynamics were also well maintained and the patient was pain-free. His visual analog scale (VAS) scores were not more than 3/10. The rest of the hospital stay was uneventful.

## Discussion

HCM is a complex cardiac condition with distinctive pathophysiologic features, most frequently presenting with hypertrophy of the interventricular septum and the anterolateral free wall of the heart. The major pathophysiologic determinants include hypertrophy of the myocardium, dynamic LVOT obstruction, mitral regurgitation caused by systolic anterior movement of the mitral valve, diastolic dysfunction, myocardial ischemia, and dysrhythmia [4]. It may present with dyspnea on exertion, angina, dizziness, presyncope, syncope, and sudden death [5]. Medical management of HCM consists of beta blockers and calcium channel blockers. Beta blockers induce negative inotropism by prolonging diastolic filling time, reducing LVOT obstruction, and reducing myocardial oxygen requirement. Atrial fibrillation and flutter are commonly associated with HCM, leading to an increased risk of arterial thromboembolism that indicates the use of anticoagulants [6].

The anesthetic goal for a patient with HCM for noncardiac surgery is to minimize LVOT obstruction, arrhythmias, and diastolic dysfunction. This can be achieved by maintenance of decreasing myocardial contractility and maintaining preload or afterload [4,7]. On the other hand, sympathetic stimulation, hypovolemia, and vasodilation should be avoided, as these may increase the LVOT gradient [5]. It becomes essential to maintain sinus rhythm as preload is mainly dependent on atrial contraction. Hence, special precautions should be taken during induction, intubation, positioning, and extubation to prevent sympathetic surges that may lead to arrhythmias, LVOT obstruction, and increased myocardial oxygen demand. Strict vigilance should be maintained to prevent all the above factors that may increase LVOT obstruction [7-9].

The sphenopalatine ganglion is a parasympathetic ganglion located within the pterygopalatine fossa of the skull with both sensory and autonomic functions [10]. Sphenopalatine ganglion blockade plays an important role in the treatment of a number of acute as well as chronic painful conditions [10]. In patients with HCM, pain and surgical stress can lead to an increase in LVOT obstruction and can precipitate hemodynamic collapse. It can complicate the perioperative course [11]. Thus, adequate analgesia with the help of peripheral nerve blocks plays a vital role in minimizing the catecholamine surge [12,13]. Sphenopalatine ganglion block can be an alternative in conjugation with other multimodal analgesia techniques in preventing hemodynamic perturbations. The main aim of administering sphenopalatine block is to decrease sympathetic activity to reduce chronotropy and inotropy. It will aid in the maintenance of afterload, heart rate, and sinus rhythm. Moreover, due to the stress and pain relief, the risk of thyroid crisis can be reduced in such hyperthyroid patients.

Endonasal orbital decompression surgery itself has a few anesthesia considerations that have to be taken care of [1]. These may include insertion of a throat pack, chances of oculocardiac reflex, and requirement of a relatively clear surgical field. In our case, the administration of sphenopalatine ganglion blockade provided adequate analgesia preventing any hemodynamic surges and thus assisting in providing the surgeons a satisfactory surgical field.

## Conclusions

Patients with HCM, when exposed to the stress of anesthesia and surgery, can experience perioperative hemodynamic complications such as congestive cardiac failure, myocardial ischemia, diastolic dysfunction, and arrhythmias. Therefore, careful intraoperative vigilance and hemodynamic monitoring are of utmost importance in such patients posted for non-cardiac surgery. Regional anesthetic block prevents the sympathetic surgery, and should therefore be considered an analgesic option as indicated especially in patients with underlying cardiac pathologies. Although there are few cases that reported successful management of HCM patients for non-cardiac surgery using peripheral nerve block, our case is the first case of using sphenopalatine block in HCM patients undergoing orbital decompression surgery, which successfully helped in maintaining analgesia and hemodynamic stability.
